# Synergistic effects of influenza and 1-methyl-4-phenyl-1,2,3,6-tetrahydropyridine (MPTP) can be eliminated by the use of influenza therapeutics: experimental evidence for the multi-hit hypothesis

**DOI:** 10.1038/s41531-017-0019-z

**Published:** 2017-05-23

**Authors:** Shankar Sadasivan, Bridgett Sharp, Stacey Schultz-Cherry, Richard Jay Smeyne

**Affiliations:** 1 Department of Developmental Neurobiology, St. Jude Children’s Research Hospital, 262 Danny Thomas Place,, Memphis, TN 38105 USA; 2 Department of Infectious Diseases, St. Jude Children’s Research Hospital, 262 Danny Thomas Place,, Memphis, TN 38105 USA; 3 Department of Neurosciences, Thomas Jefferson University, 233 South 10th Street,, Philadelphia, PA 19107 USA

## Abstract

Central Nervous System inflammation has been implicated in neurodegenerative disorders including Parkinson’s disease (Ransohoff, Science 353: 777–783, 2016; Kannarkat et al. J. Parkinsons Dis. 3: 493–514, 2013). Here, we examined if the H1N1 influenza virus (Studahl et al. Drugs 73: 131–158, 2013) could synergize with the parkinsonian toxin 1-methyl-4-phenyl-1,2,3,6-tetrahydropyridine (Jackson-Lewis et al. in Mark LeDoux (ed) *Movement Disorders: Genetics and Models*: 287–306, Elsevier, 2015) to induce a greater microglial activation and loss of substantia nigra pars compacta dopaminergic neurons than either insult alone. H1N1-infected animals administered 1-methyl-4-phenyl-1,2,3,6-tetrahydropyridine exhibit a 20% greater loss of substantia nigra pars compacta dopaminergic neurons than occurs from the additive effects of H1N1 or 1-methyl-4-phenyl-1,2,3,6-tetrahydropyridine alone (*p* < 0.001). No synergistic effects were found in microglial activation. The synergistic dopaminergic neuron loss is eliminated by influenza vaccination or treatment with oseltamivir carboxylate. This work shows that multiple insults can induce synergistic effects; and even these small changes can be significant as it might allow one to cross a phenotypic disease threshold that would not occur from individual non-interacting exposures. Our observations also have important implications for public health, providing impetus for influenza vaccination or prompt treatment with anti-viral medications upon influenza diagnosis.

Influenza A viruses infect a number of different species, ranging from birds to mammals including humans.^1^ In addition to the well-defined respiratory effects, acute influenza infection in humans can lead to the development of a number of encephalitic syndromes, each having neurological consequences.^2^ We demonstrated that acute infection in mice with two different influenza viruses, A/Vietnam/1203/2004 (highly pathogenic avian H5N1 virus)^3^ and A/California/04/2009 H1N1 virus,^4^ induces an inflammatory response in the brain, consisting of activation of microglia and secretion of cytokines/chemokines. This neuroinflammatory response was independent of viral neurotropism as the H1N1 virus is not neurotropic in mice.^3, 4^ This suggested that the peripheral immune response activated following influenza infection^5, 6^ was likely responsible for the observed secondary Central Nervous System (CNS) inflammation.

Of concern, inflammation within the CNS can increase the sensitivity to secondary insults, such as agents that induce proteasome inhibition or induce oxidative stress, that otherwise would not induce significant neurological damage.^7–11^ To test if influenza virus could act as one of the insults in the “multi-hit” hypothesis,^12^ we used the optical disector method (Stereoinvestigator, MBF Biosciences, Williston, VT) to estimate the number of activated microglia^4^ (as a marker of neuroinflammation) in the substantia nigra pars compacta (SNpc) and model-based stereology^13^ to estimate the number of dopaminergic (DA) neurons (TH-positive + TH-negative, Nissl-positive DA neurons)^14^ in four groups of animals: (1) mice intranasally administered saline; (2) mice intranasally administered 10^2^ TCID_50_ influenza H1N1 virus^4^; (3) mice administered 4 × 20 mg/kg 1-methyl-4-phenyl-1,2,3,6-tetrahydropyridine (MPTP)^4^; and (4) mice intranasally administered 10^2^ TCID_50_ influenza H1N1 virus followed 30 days later by 4 × 20 mg/kg MPTP. All conditions were blinded prior to counting. All of the experiments were approved by the SJCRH (protocol 513) and TJU (protocol 1892) IACUCs and performed in accordance with the NIH Guide for the Care and Use of Laboratory Animals. The number of activated microglia and SNpc DA in each condition were subsequently compared using one-way ANOVA followed by post hoc Bonferroni comparisons (Prism 7 for Mac, GraphPad Software).

We found increased numbers of activated microglia in the SNpc (Fig. 1a) 7 days after MPTP (345% increase vs. control, *p* < 0.0002) or 30 days after H1N1 (231% increase vs. control, *p* < 0.01), supporting our previous observations.^4, 15^ When mice were exposed to H1N1, and then 30 days later to MPTP, we observed no additional increase in the number of activated microglia compared with the MPTP group (decrease of 7%, NS), suggesting either: (1) that there was no combinatorial (either additive (x + y = z) or synergistic (x + y < z)) inflammatory effect of H1N1 + MPTP on (2) microglial activation or that MPTP induced a maximal microglial activation (Fig. 1a). Unlike the activation of microglia, we found that the SNpc DA neuron loss was 20% greater (47% loss compared with saline, *p* < 0.0001) than the additive neuronal loss induced by H1N1 (8% loss compared with saline, NS) or MPTP (26% loss compared with saline, *p* < 0.001) alone, suggesting a significant synergistic effect in DA neuron loss, where SNpc DA neuron loss was increased by 25% in the influenza infected + MPTP animals as compared with MPTP (*p* < 0.05) or H1N1 (*p* < 0.0001) alone (Fig. 1b). This demonstrated that prior influenza infection, even if resolved, could increase the sensitivity of DA neurons to a second insult.

**Fig. 1:**
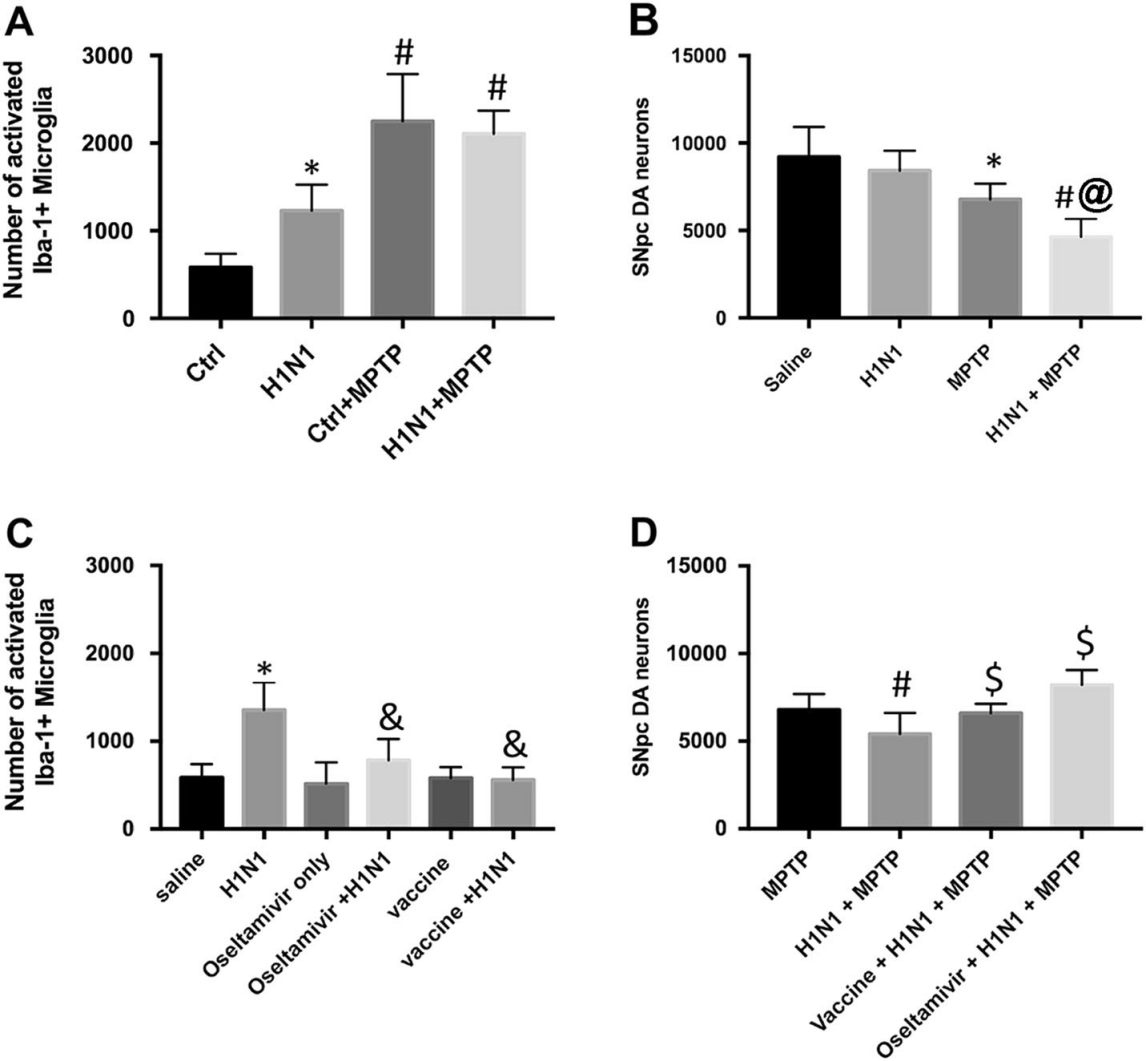
Effects of H1N1 and MPTP on microglia and DA neurons in the SNpc. **a** Stereological estimate of the number of activated microglia in the SNpc 30 days after saline (control, *n* = 15), 30 days after H1N1 (*n* = 7), 7 days after MPTP (*n* = 7), or 7 days after H1N1 + MPTP (*n* = 10). **b** Stereological estimate of the number of DA neurons in the SNpc 30 days after H1N1, MPTP, or H1N1 + MPTP. **c** Stereological estimate of the number of activated microglia in the SNpc 30 days after H1N1, H1N1 + oseltamivir (*n* = 10), or H1N1 + vaccine (*n* = 7). **d** Stereological estimate of the number of DA neurons in the SNpc 30 days after MPTP, H1N1 + MPTP, H1N1 + MPTP + oseltamivir, or H1N1 + MPTP + vaccine. ^*^ *p* < 0.05 compared with control, ^#^ *p* < 0.05 compared with H1N1 or control, ^&^ *p* < 0.05 compared with H1N1, ^$^ *p* < 0.05 compared with H1N1 + MPTP, ^@^ *p* < 0.05 compared with MPTP or H1N1 alone

Having shown that H1N1 influenza infection can act as the first “hit” in a “multi-hit” model,^16^ we examined whether two different influenza therapeutics could eliminate this synergy. Here, we either intramuscularly vaccinated mice with an inactivated strain-matched H1N1 influenza virus vaccine 30 days prior to H1N1 infection or orally administered oseltamivir carboxylate, a neuraminidase inhibitor that has been shown to be 99.2% effective against H1N1pdm09 viruses,^17^ twice a day for 6 days beginning 1 day before influenza virus infection. In both paradigms, we allowed 30 days to elapse and then quantitated the number of SNpc DA neurons and activated microglia. We found that administration of either treatment completely alleviated the increase in H1N1 virus-induced microglial activation (Fig. 1c), but did not reduce the microglial activation induced by MPTP alone (data not shown). We then asked if influenza therapeutics could alter the synergy seen by H1N1 + MPTP, as it is related to SNpc DA neuron loss. Here we either vaccinated mice prior to H1N1 infection or treated with oseltamivir carboxylate concomitant to H1N1 infection as described above. After 30 days, these mice were administered 4 × 20 mg/kg MPTP and 7 days later we estimated the number of SNpc DA neurons. In both the vaccine-treated (6590 ± 220) and oseltamivir-treated mice (7221 ± 266), H1N1 + MPTP induced SNpc DA loss that was statistically identical to animals administered MPTP-only (Fig. 1d). This suggested that alleviating the inflammatory program induced by H1N1 infection by either vaccination or oseltamivir carboxylate could abrogate the increased SNpc DA neuron death induced by a second “hit” seen without influenza intervention.

The majority of Parkinson’s disease cases have an unknown etiology, although it is generally thought that it results from an interaction of environmental insult(s) with an underlying susceptibility to these agents.^18, 19^ Since Parkinson’s disease generally manifests starting in the sixth decade of life,^20^ it is likely that any person will encounter any number of environmental insults, which alone may be innocuous but together may synergize to produce a measureable pathology. Our results suggest that influenza-induced activation of the intrinsic immune system of the brain can exacerbate the effects of a known parkinsonian agent, MPTP. From 2009 to 2013, approximately 24% of the worldwide population had been exposed to the H1N1 virus^21^ and its re-emergence in subsequent years likely increased these numbers. If these preclinical studies translate to humans, the results from this study suggest that influenza infection could be a common risk factor for sensitizing the SNpc DA neurons to oxidative stress and subsequent development of parkinsonism. Here, we show that the synergistic effects of the “influenza (hit 1) × oxidative-stress induction (hit 2)” interaction can be eliminated by vaccination or prompt treatment with the neuraminidase inhibitor oseltamivir carboxylate, suggesting that prevention of the H1N1-induced microglial activation can mitigate this potential parkinsonian risk factor.

In the 6-year period prior to 2015 it has been estimated that 60% of the US population (based on a US Census estimated at 324 million persons) has been vaccinated against influenza.^22^ In the last full year (2014–2015) in which statistics had been released, approximately 692,000 tests were performed for flu during physician visits for influenza-like symptoms, of which 18% were positive.^23^ Additionally, other studies suggest that about 13% of physician visits for influenza-like symptoms result in prescription of an anti-viral medication.^24^ Using these figures, there is a large number of persons (approximately 190 million) who are potentially exposed to flu but go untreated, each, based on these preclinical studies, of whom would have the potential to be at increased risk for developing Parkinson’s disease. Despite the lack of a defined mechanism and studies examining the maximal interval in which these “hits” can interact—both of which warrant further study—our observations have potentially important implications in public health and provide additional impetus for influenza vaccination or prompt treatment with anti-viral medications upon influenza diagnosis.

## References

[1] Yoon SW, Webby RJ, Webster RG (2014). Evolution and ecology of influenza A viruses. Curr. Top. Microbiol. Immunol..

[2] Studahl M (2003). Influenza virus and CNS manifestations. J. Clin. Virol..

[3] Jang H (2012). Inflammatory effects of highly pathogenic H5N1 influenza virus infection in the CNS of mice. J. Neurosci..

[4] Sadasivan S, Zanin M, O’Brien K, Schultz-Cherry S, Smeyne RJ (2015). Induction of microglia activation after infection with the non-neurotropic A/CA/04/2009 H1N1 influenza virus. PLoS ONE.

[5] Rezai-Zadeh K, Gate D, Town T (2009). CNS infiltration of peripheral immune cells: D-day for neurodegenerative disease?. J. Neuroimmune Pharmacol..

[6] Liu Q, Zhou YH, Yang ZQ (2016). The cytokine storm of severe influenza and development of immunomodulatory therapy. Cell Mol. Immunol..

[7] de Pablos RM (2014). Chronic stress enhances microglia activation and exacerbates death of nigral dopaminergic neurons under conditions of inflammation. J. Neuroinflammat..

[8] Tansey MG (2010). Inflammation in neuropsychiatric disease. Neurobiol. Dis..

[9] Pintado C (2012). Lipopolysaccharide-induced neuroinflammation leads to the accumulation of ubiquitinated proteins and increases susceptibility to neurodegeneration induced by proteasome inhibition in rat hippocampus. J. Neuroinflammat..

[10] Koprich JB, Reske-Nielsen C, Mithal P, Isacson O (2008). Neuroinflammation mediated by IL-1beta increases susceptibility of dopamine neurons to degeneration in an animal model of Parkinson’s disease. J. Neuroinflammat..

[11] Kanaan NM, Kordower JH, Collier TJ (2008). Age and region-specific responses of microglia, but not astrocytes, suggest a role in selective vulnerability of dopamine neurons after 1-methyl-4-phenyl-1,2,3,6-tetrahydropyridine exposure in monkeys. Glia.

[12] Carvey PM, Punati A, Newman MB (2006). Progressive dopamine neuron loss in Parkinson’s disease: the multiple hit hypothesis. Cell Transplant..

[13] Baquet ZC, Williams D, Brody J, Smeyne RJ (2009). A comparison of model-based (2D) and design-based (3D) stereological methods for estimating cell number in the substantia nigra pars compacta (SNpc) of the C57BL/6J mouse. Neuroscience.

[14] Hamre K, Tharp R, Poon K, Xiong X, Smeyne RJ (1999). Differential strain susceptibility following 1-methyl-4-phenyl-1,2,3,6-tetrahydropyridine (MPTP) administration acts in an autosomal dominant fashion: quantitative analysis in seven strains of Mus musculus. Brain Res..

[15] Smeyne, R. J. et al. Assessment of the effects of MPTP and paraquat on dopaminergic neurons and microglia in the substantia nigra pars compacta of C57BL/6 mice. *PLoS ONE*. **11**, e0164094 (2016). 10.1371/journal.pone.0164094 PMC5082881 27788145

[16] Sulzer D (2007). Multiple hit hypotheses for dopamine neuron loss in Parkinson’s disease. Trends Neurosci..

[17] Centers for Disease Control. Influenza Antiviral Drug Resistance. Centers for Disease Control and Prevention, National Center for Immunization and Respiratory Diseases (NCIRD). (2016).

[18] Boger HA, Granholm AC, McGinty JF, Middaugh LD (2010). A dual-hit animal model for age-related parkinsonism. Prog. Neurobiol..

[19] Gao HM, Hong JS (2011). Gene-environment interactions: key to unraveling the mystery of Parkinson’s disease. Prog. Neurobiol..

[20] Elbaz A (2002). Risk tables for parkinsonism and Parkinson’s disease. J. Clin. Epidemiol..

[21] Van Kerkhove MD, Hirve S, Koukounari A, Mounts AW (2013). Estimating age-specific cumulative incidence for the 2009 influenza pandemic: a meta-analysis of A(H1N1)pdm09 serological studies from 19 countries. Influenza Other Respir. Viruses.

[22] Santibanez, T. A. et al. Flu Vaccination Coverage, United States, 2014–15 Influenza Season. (2016).

[23] Appiah GD (2015). Influenza activity—United States, 2014–15 season and composition of the 2015-16 influenza vaccine. Morb. Mortal. Wkly. Rep..

[24] Atkins CY (2011). Estimating effect of antiviral drug use during pandemic (H1N1) 2009 outbreak, United States. Emerg. Infect. Dis..

